# Transcranial Doppler ultrasound in evaluating cerebral blood flow abnormalities in major depressive disorder

**DOI:** 10.1097/MD.0000000000039889

**Published:** 2024-10-18

**Authors:** Kailin Gong, Yuting Li, Junfei Rong, Jiajia Song, Fangfang Ren

**Affiliations:** a Department of Physical Diagnosis, Brain Hospital Affiliated to Nanjing Medical University, Nanjing, Jiangsu, China; b Department of Psychiatry, Brain Hospital Affiliated to Nanjing Medical University, Nanjing, Jiangsu, China; c Jiangsu Prison Administration Jiangbei Hospital Outpatient Department, Nanjing, Jiangsu, China.

**Keywords:** blood flow velocity, depression, Hamilton Depression Rating Scale, middle cerebral artery, Montgomery–Åsberg Depression Rating Scale, pulsatility index, resistance index

## Abstract

Previous research has shown that blood flow abnormalities affect major depressive disorder (MDD) from multiple perspectives. Therefore, this study aims to investigate the relationship between middle cerebral artery (MCA) blood flow velocity parameters and clinical symptom scores (Hamilton Depression Rating Scale [HAMD] and Montgomery–Åsberg Depression Rating Scale [MADRS]) in patients with MDD. We compared the MCA blood flow velocity parameters, including peak systolic velocity (MCA-PSV), end-diastolic velocity (MCA-EDV), and mean velocity (MCA-MV), between 50 MDD patients and 50 control subjects. Additionally, we analyzed the correlation between these parameters and HAMD and MADRS scores. Hemodynamic parameters such as pulsatility index and resistance index were also compared between the 2 groups. MCA-PSV, MCA-EDV, and MCA-MV were significantly lower in MDD patients compared to the control group, while pulsatility index and resistance index were significantly higher. Correlation analysis revealed that MCA-PSV, MCA-EDV, and MCA-MV were significantly negatively correlated with HAMD and MADRS scores in MDD patients, indicating that cerebral blood flow velocity decreases as depressive symptoms worsen. Furthermore, regression analysis confirmed the negative relationship between blood flow velocity parameters and clinical symptom scores. The results of this study suggest that the reduction in cerebral blood flow velocity in MDD patients may be associated with the severity of depressive symptoms. This finding provides new insights into the pathophysiological mechanisms of MDD and offers a potential theoretical basis for developing depression treatment strategies based on cerebral blood flow velocity parameters.

## 1. Introduction

Depression, as a global mental health issue, has gradually become a significant disease affecting the quality of life and psychological health of people worldwide.^[1–3]^ Major depressive disorder (MDD), a severe form of depression, is characterized by prominent symptoms such as persistent low mood, loss of interest, and decreased energy, causing substantial distress to patients and severely impacting their social functioning and daily lives.^[4–6]^ With the advancement of medical research, increasing evidence suggests that the onset of depression is closely related to abnormalities in brain function and structure, particularly changes in cerebral blood flow, which play a crucial role in the pathogenesis of depression.^[7,8]^

Cerebral blood flow is the primary means by which the brain obtains energy and nutrients, and it is essential for maintaining normal brain function.^[9,10]^ In patients with depression, abnormal changes in cerebral blood flow can lead to insufficient energy supply to neurons and may affect the synthesis and release of neurotransmitters, further exacerbating depressive symptoms.^[11,12]^ In addition, studies have shown that the main consequences of blood flow disorders are cognitive deficits, such as decreased executive function, revealing the relationship between cortical activity and cerebral blood flow during patient active control, which may also increase depressive symptoms and be associated with functional disability, poor quality of life, increased risk of relapse after recovery, and severity of residual symptoms^[13–16]^. Therefore, accurately assessing the cerebral blood flow status of patients with depression is of great importance for understanding its pathogenesis and guiding clinical treatment.

Most previous studies have examined regional cerebral blood flow abnormalities in major depressive disorder (MDD), usually by injecting radioisotopes, and identified abnormalities in regional cerebral blood flow index that differ from normal in MDD patients. However, methods such as radioisotope or arterial spin labeling magnetic resonance imaging increase the risk of radiation and allergies, are expensive, and carry the risk of overtreatment. Transcranial Doppler ultrasonography (TCD), as a noninvasive, convenient, and repeatable technique for detecting cerebral blood flow, has gained widespread application in the field of neuroscience in recent years.^[17–19]^ This technique involves using an ultrasound probe to emit ultrasonic waves into the cranium and monitoring the blood flow velocity and hemodynamic parameters of intracranial vessels in real-time based on the scattering and reflection properties of the ultrasonic waves in the blood. Compared to traditional methods of assessing cerebral blood flow, TCD has advantages such as noninvasiveness, no need for radioactive substances, and the ability to perform real-time monitoring, making it particularly suitable for evaluating cerebral blood flow in mental disorders such as depression.^[20,21]^ TCD has the advantage of real-time monitoring and noninvasive in evaluating cerebral blood flow in MDD, which helps to detect abnormal changes and conduct long-term monitoring.^[21]^

However, the indicators of Doppler ultrasound are not fully utilized at present, mainly because there are few studies on the correlation between Doppler ultrasound cerebral blood flow data and major depression and whether there is predictive value. However, based on previous experience information, we believe that Doppler ultrasound blood flow data may have a certain correlation and predictability in patients with major depression. Therefore, this study aims to explore the role of TCD in evaluating cerebral blood flow abnormalities in MDD. We will use TCD to assess cerebral blood flow in a group of MDD patients and compare the results with a healthy control group. By comparing the differences in cerebral blood flow velocity and hemodynamic parameters between the 2 groups, we aim to uncover the characteristics and patterns of cerebral blood flow abnormalities in MDD patients and investigate their correlation with depressive symptoms. Additionally, we will conduct a preliminary exploration of the standardized operational methods and evaluation criteria for TCD in the assessment of cerebral blood flow in depression, aiming to provide more accurate and reliable data for clinical treatment and efficacy evaluation. This study not only contributes to a deeper understanding of the pathogenesis of MDD but also provides new ideas and methods for clinical diagnosis and treatment. We believe that with ongoing research and technological advancements, TCD will play a more significant role in the field of neuroscience and make greater contributions to human health.

## 2. Materials and methods

### 2.1. Study design

This retrospective study was approved by the Ethics Committee of the Brain Hospital Affiliated to Nanjing Medical University. By comparing the cerebral blood flow velocity and hemodynamic parameters of MDD patients with those of healthy controls, this study aims to reveal the characteristics of cerebral blood flow abnormalities in MDD patients and explore their relationship with clinical symptoms.

### 2.2. Sample selection and sample size

(1) Sample selection: this study included a total of 100 participants, comprising 50 MDD patients and 50 healthy controls. MDD patients were required to meet the diagnostic criteria for MDD according to the International Classification of Diseases (ICD-10) or the Diagnostic and Statistical Manual of Mental Disorders (DSM-5), with symptoms persisting for at least 2 weeks and severely impacting daily life. Healthy controls were required to have no history of mental illness, be matched with MDD patients in age and gender, and have no other conditions that might affect cerebral blood flow. Information about patients in the healthy control group was derived from physical examinations.
(2) Sample size calculation: the determination of the sample size was based on a review of prior literature and preliminary experimental results, anticipating significant differences in cerebral blood flow parameters between MDD patients and healthy controls. Setting *α* = 0.05 (two-tailed) and an effect size of 0.5 (medium effect), and considering a 20% dropout rate, the sample size calculation software indicated that at least 42 participants were needed in each group. To ensure the reliability of the study, we ultimately decided to include 50 participants in each group.

### 2.3. Inclusion and exclusion criteria

(1) Inclusion criteria:
  - MDD group: participants who met the International Classification of Diseases (ICD-10) or Diagnostic and Statistical Manual of Mental Disorders (DSM-5) diagnostic criteria for MDD, aged 18 to 65, and voluntarily participated in the study with informed consent.
  - Control group: participants with no history of mental illness, matched in age and gender with the MDD group, and voluntarily participated in the study with informed consent.
(2) Exclusion criteria:
  - Individuals with other mental or neurological disorders.
  - Individuals who had recently (within 1 month) used medications that might affect cerebral blood flow.
  - Pregnant or lactating women.
  - Individuals with severe physical or organic diseases.
  - Individuals unable to cooperate with the TCD examination.

### 2.4. Detection indicators

(1) Cerebral blood flow velocity: including peak systolic velocity (PSV), end-diastolic velocity (EDV), and mean velocity (MV) of the middle cerebral artery (MCA).
(2) Hemodynamic parameters: including pulsatility index (PI), resistance index (RI), and blood flow spectral characteristics.
(3) Clinical symptom assessment: using the Hamilton Depression Rating Scale (HAMD) and Montgomery–Åsberg Depression Rating Scale (MADRS) to quantitatively assess the depressive symptoms of MDD patients.

### 2.5. General subject information

There were no significant differences in age and gender distribution between the MDD group and the control group (age: MDD group 38.2 ± 10.5 years vs control group 37.8 ± 9.7 years; gender: MDD group 23 males/27 females vs control group 22 males/28 females). However, the duration of illness was significantly longer in the MDD group compared to the control group (MDD group 18.3 ± 8.2 months vs control group no data). Additionally, HAMD scores (24.6 ± 5.3) and MADRS scores (32.1 ± 7.4) were significantly higher in the MDD group compared to the control group (HAMD score: 3.1 ± 1.2; MADRS score: 4.2 ± 1.5), indicating the presence of significant depressive symptoms in the MDD group (Table 1).

**Table 1:** Demographic characteristics of subjects.

Group	MDD	Control	T/X^2^	*P*-value
Number	50	50		
Age (years)	38.2 ± 10.5	37.8 ± 9.7	1.02	.124
Gender (male/female)	23/27	22/28	0.04	.841
Duration of illness (months)	18.3 ± 8.2	–	–	–
HAMD score	24.6 ± 5.3	3.1 ± 1.2	6.21	<.001
MADRS score	32.1 ± 7.4	4.2 ± 1.5	8.53	<.001

*Note*: Age is presented as mean ± standard deviation; duration of illness refers to the duration from the onset of symptoms to enrolment in the MDD group; HAMD and MADRS scores are presented as mean ± standard deviation.

### 2.6. Detection methods

TCD examination^[22]^: high-resolution TCD equipment with a 2 MHz probe was used. During the examination, participants were required to remain quiet and relaxed. The probe was placed on both sides of the temporal windows and the occipital window to detect blood flow signals in the bilateral MCA. Parameters such as PSV, EDV, MV, PI, and RI were recorded, and the blood flow spectral characteristics were observed. All examinations were performed by experienced sonographers.

Clinical symptom assessment^[23]^: professional psychologists conducted HAMD and MADRS assessments for MDD patients to quantify the severity of their depressive symptoms. During the assessment, it was ensured that participants fully understood the scale content and answered the relevant questions truthfully.

### 2.7. Statistical methods

In this study, we strictly followed scientific statistical methods to ensure the accuracy and reliability of the results. Firstly, data entry and organization were precisely completed using Excel or professional statistical software to ensure the accuracy and completeness of all raw data. Next, we performed detailed descriptive statistical analysis on the general data and detection indicators (such as PSV, EDV, MV, PI, and RI of the MCA), including key indicators such as mean, standard deviation, median, and interquartile range.

To compare the differences in cerebral blood flow velocity and hemodynamic parameters between the MDD group and the control group, we used independent samples *t* tests or Mann–Whitney *U* tests, depending on the distribution of the data. Additionally, chi-square tests were used to compare categorical variables such as gender between the 2 groups.

In exploring the relationship between cerebral blood flow parameters and clinical symptom scores in MDD patients, we employed Spearman or Pearson correlation analysis, selecting the most appropriate correlation analysis method based on the specific characteristics and distribution of the data. These analyses helped us understand the potential links between cerebral blood flow parameters and clinical symptoms.

To further investigate the primary factors influencing cerebral blood flow in MDD patients, we conducted multiple linear regression analysis, using cerebral blood flow parameters as independent variables and clinical symptom scores as dependent variables. This step enabled us to gain a deeper understanding of the specific impact of various factors on patients’ cerebral blood flow.

Throughout the statistical analysis process, we used SPSS 26.0 or higher versions of the statistical software, which provides rich functionalities and powerful computing capabilities for data analysis and processing. All significance levels for statistical analyses were set at *α* = 0.05 to ensure the reliability and rigor of the results.

## 3. Results

This study compared cerebral blood flow velocity parameters, hemodynamic parameters, and the correlation between these parameters and clinical symptom scores between the MDD group and the control group. The following is a detailed overview of the research results.

### 3.1. Comparison of cerebral blood flow velocity parameters

The MDD group exhibited significantly lower peak systolic velocity (MCA-PSV), end-diastolic velocity (MCA-EDV), and mean velocity (MCA-MV) in the middle cerebral artery compared to the control group (MCA-PSV: MDD group 53.2 ± 11.8 cm/s vs control group 61.5 ± 10.3 cm/s; MCA-EDV: MDD group 19.6 ± 5.2 cm/s vs control group 25.1 ± 4.8 cm/s; MCA-MV: MDD group 31.4 ± 6.7 cm/s vs control group 37.2 ± 5.9 cm/s). These results indicate that cerebral blood flow velocity in the middle cerebral artery is reduced in MDD patients compared to the control group (Table 2).

**Table 2:** Comparison of cerebral blood flow velocity parameters.

Group	MDD	Control	t-value	*P*-value
MCA-PSV (cm/s)	53.2 ± 11.8	61.5 ± 10.3	–4.2	<.001
MCA-EDV (cm/s)	19.6 ± 5.2	25.1 ± 4.8	–6.5	<.001
MCA-MV (cm/s)	31.4 ± 6.7	37.2 ± 5.9	–5.3	<.001

*Note*: MCA-PSV, MCA-EDV, and MCA-MV represent the peak systolic velocity, end-diastolic velocity, and mean velocity of the middle cerebral artery, respectively. All values are presented as mean ± standard deviation.

### 3.2. Comparison of hemodynamic parameters

The MDD group had higher PI and RI compared to the control group (PI: MDD group 1.21 ± 0.15 vs control group 0.98 ± 0.12; RI: MDD group 0.72 ± 0.08 vs control group 0.65 ± 0.07). The increase in these parameters may reflect increased cerebral vascular resistance and altered hemodynamics in MDD patients (Table 3).

**Table 3:** Comparison of hemodynamic parameters.

Group	MDD	Control	t-value	*P*-value
PI	1.21 ± 0.15	0.98 ± 0.12	7.8	<.001
RI	0.72 ± 0.08	0.65 ± 0.07	5.1	<.001

*Note*: PI represents the pulsatility index, and RI represents the resistance index. All values are presented as mean ± standard deviation.

### 3.3. Correlation analysis between cerebral blood flow velocity and clinical symptom scores

Correlation analysis showed that cerebral blood flow velocity parameters (MCA-PSV, MCA-EDV, and MCA-MV) in the MDD group were significantly negatively correlated with HAMD scores and MADRS scores (*P* < .05). This indicates that as depressive symptoms worsen, the cerebral blood flow velocity in patients decreases accordingly (Table 4).

**Table 4:** Correlation analysis between cerebral blood flow velocity and clinical symptom scores.

Blood flow velocity parameter	HAMD score	MADRS score
MCA-PSV	−0.52*	−0.48*
MCA-EDV	−0.45*	−0.41*
MCA-MV	−0.50*	−0.46*

* A significant negative correlation between cerebral blood flow velocity and clinical symptom scores at the *α* = 0.05 level.

### 3.4. Regression analysis results

Regression analysis further confirmed the negative relationship between cerebral blood flow velocity parameters (MCA-PSV, MCA-EDV, and MCA-MV) and HAMD scores and MADRS scores. Specifically, decreases in MCA-PSV, MCA-EDV, and MCA-MV can predict increases in HAMD and MADRS scores, indicating a worsening of depressive symptoms. This finding provides new evidence for understanding the relationship between changes in cerebral blood flow and depressive symptoms in MDD patients (Table 5).

**Table 5:** Regression analysis results.

Independent variable	Dependent variable	β-value	t-value	*P*-value
MCA-PSV	HAMD score	−0.43	−3.98	<.001
MCA-EDV	HAMD score	−0.31	−2.86	.006
MCA-MV	HAMD score	−0.4	−3.69	<.001
MCA-PSV	MADRS score	−0.38	−3.51	<.001
MCA-EDV	MADRS score	−0.29	−2.68	.009
MCA-MV	MADRS score	−0.36	−3.31	.001

*Note*: β-value represents the regression coefficient, t-value is the statistic for the *t* test, and *P*-value represents the significance level.

### 3.5. ROC analysis results

ROC analysis results explored the prediction of cerebral blood flow parameters on HAMD score and MARDS score, and it could be seen that the overall prediction was above 0.6. Specifically, the prediction ability of each individual cerebral blood flow parameter to increase the risk of HAMD score and MARDS score was above 60% probability. In particular, when MCA-EDV blood flow parameter increased by 1, the risk of HAMD score was reduced by 0.762 times, which was the blood flow parameter with the best predictive ability, as shown in Table 6.

**Table 6:** ROC analysis results.

Independent variable	Dependent variable	AUC	95%CI	Standard error	*P*-value
MCA-PSV	HAMD score	0.653	0.569–0.737	0.043	<.001
MCA-EDV	HAMD score	0.762	0.698–0.826	0.033	<.001
MCA-MV	HAMD score	0.703	0.621–0.785	0.042	<.001
MCA-PSV	MADRS score	0.624	0.550–0.698	0.038	<.001
MCA-EDV	MADRS score	0.751	0.691–0.811	0.031	<.001
MCA-MV	MADRS score	0.716	0.661–0.771	0.028	<.001

*Note*: *P*-value < .05 represents the significance level.

## 4. Discussion

This study explored the characteristics of cerebral blood flow abnormalities and their relationship with clinical symptoms in MDD patients through TCD and clinical symptom assessments in 50 MDD patients and 50 healthy controls. The following discussion elaborates on the study results from multiple aspects.

### 4.1. Characteristics of cerebral blood flow abnormalities in MDD patients

The data from Tables 2 and 3 show that the cerebral blood flow velocity parameters (including MCA-PSV, MCA-EDV, and MCA-MV) were significantly lower in MDD patients compared to healthy controls, while PI and RI were significantly higher. This indicates that MDD patients exhibit reduced blood flow velocity and increased resistance in the middle cerebral artery, reflecting notable cerebral blood flow abnormalities. Several factors may contribute to the cerebral blood flow abnormalities observed in MDD patients. First, MDD patients often experience autonomic nervous system dysfunction, which can lead to abnormalities in cerebrovascular vasomotion, thereby affecting cerebral blood flow velocity. Second, there may be imbalances in neurotransmitters such as serotonin and norepinephrine in MDD patients, which are crucial for regulating cerebrovascular function; such imbalances might result in cerebral blood flow abnormalities. Additionally, MDD patients frequently suffer from poor sleep, irregular eating habits, and other adverse lifestyle factors that might also impact cerebral blood flow.^[12,23–25]^

### 4.2. Relationship between cerebral blood flow abnormalities and clinical symptoms in MDD

The data from Table 4 indicate a significant negative correlation between cerebral blood flow velocity and scores on the HAMD and MADRS in MDD patients. This suggests that cerebral blood flow abnormalities might serve as an important biological marker for clinical symptoms of MDD.^[7,26,27]^ The relationship between cerebral blood flow abnormalities and clinical symptoms of MDD might involve several mechanisms. First, abnormalities in cerebral blood flow may lead to metabolic disturbances in brain neurons, affecting the synthesis and release of neurotransmitters, thereby exacerbating MDD symptoms. Second, cerebral blood flow abnormalities could cause localized brain ischemia or hypoxia, leading to neuronal damage or apoptosis, further intensifying the pathological process of MDD. Additionally, cerebral blood flow abnormalities might disrupt brain functional connectivity and information transmission, resulting in cognitive dysfunction and emotional regulation issues in MDD patients.^[28,29]^ The study found that in people with major depression, blood flow to the brain was associated with poorer performance on the task when they saw colored words. This suggests that changes in cerebral blood flow may be related to the performance of cognitive or emotional functions in patients with depression.^[13–16]^ In addition, we conducted an independent correlation analysis of the control group, which found no significant association and therefore did not present a negative result. We believe that this may be caused by the lack of depressive mood in the control group, so the depression score does not fluctuate significantly, and the relationship between differentiation and statistical validity is not significant.

### 4.3. Significance of cerebral blood flow abnormalities in MDD treatment

The regression analysis results from Table 5 show a significant negative correlation between cerebral blood flow velocity (particularly MCA-PSV) and clinical symptom scores in MDD patients. This finding suggests that focusing on and improving cerebral blood flow abnormalities might be a critical direction for MDD treatment. Currently, MDD treatment mainly relies on pharmacotherapy and psychotherapy. However, traditional antidepressants often have slow onset and significant side effects and are ineffective for some patients. Therefore, exploring new treatment methods is essential for enhancing MDD treatment outcomes. Cerebral blood flow abnormalities, as a significant biological marker of MDD, offer a new therapeutic perspective.^[30–32]^ For example, improving cerebral blood flow abnormalities in MDD patients might accelerate the onset of antidepressants, reduce side effects, and enhance treatment efficacy. Furthermore, physical treatments (such as transcranial magnetic stimulation and transcranial direct current stimulation) or rehabilitation training targeting cerebral blood flow abnormalities could become new methods for treating MDD.

### 4.4. Future research directions

Although this study identified cerebral blood flow abnormalities in MDD patients and preliminarily explored their relationship with clinical symptoms, many questions remain unanswered and require further research. For instance, deeper investigations are needed into the mechanisms underlying cerebral blood flow abnormalities in MDD patients and the effects of different antidepressants on cerebral blood flow. Moreover, further studies should examine the relationship between cerebral blood flow abnormalities and symptoms such as cognitive dysfunction and emotional regulation disorders in MDD, as well as how to effectively target cerebral blood flow abnormalities in treatment.

In summary, this study found cerebral blood flow abnormalities in MDD patients through TCD examination and preliminarily explored their relationship with clinical symptoms. These findings not only provide new insights for the diagnosis and treatment of MDD but also offer important clues for further understanding the pathophysiological mechanisms of MDD. Future research should continue to delve into the mechanisms and treatment methods related to cerebral blood flow abnormalities in MDD patients to improve treatment outcomes and enhance patient quality of life.

## Author contributions

**Conceptualization:** Yuting Li, Junfei Rong, Jiajia Song, Fangfang Ren.

**Data curation:** Kailin Gong, Yuting Li, Junfei Rong, Fangfang Ren.

**Formal analysis:** Kailin Gong.

**Funding acquisition:** Fangfang Ren.

**Investigation:** Kailin Gong, Yuting Li, Junfei Rong, Jiajia Song, Fangfang Ren.

**Methodology:** Yuting Li, Junfei Rong, Jiajia Song, Fangfang Ren.

**Validation:** Jiajia Song.

**Visualization:** Jiajia Song.

**Writing** – **original draft:** Kailin Gong, Fangfang Ren.

**Writing** – **review & editing:** Kailin Gong, Fangfang Ren.
